# Iodine Preparations for Dental Use

**Published:** 1868-02

**Authors:** 


*Iodine Preparations for Dental Use.*—The good effects which have attended the use of Iodine and its combinations, as dental medicines, have been so great that they are now generally used in the place of some of the less effective remedies upon which the profession relied, with varying success, for many years. For local applications in alveolar abscess, alveolar periostitis, absorption of alveolar processes, &c., the following preparations are now used: Tincture of Iodine, Colorless tincture of Iodine, Tincture of Iodine and Creosote, Carbolate of Iodine.

We have lately received from Dr. S. S. White a very excellent combination of Iodine and Carbolic acid, (Carbolate of Iodine), an account of which appeared in the Decem. No. of *Am. Journal of Dental Science.* We have also received from Dr. White a preparation consisting of Elixir of Vitriol and Tamnin, for use as an astringent and hæmostatic.

